# Comparison of Different Adhesive Systems on Bond Strength of Resin Composite Posts Placed in Primary Teeth

**DOI:** 10.1155/2022/1968781

**Published:** 2022-08-29

**Authors:** Zahra Bahrololoomi, Fateme Mehravar

**Affiliations:** Department of Pedodontics, Shahid Sadoughi University of Medical Sciences, Yazd, Iran

## Abstract

**Background:**

This study evaluated the push-out bond strength of resin composite posts to the intracanal dentin in primary teeth using different adhesive systems.

**Materials and Methods:**

In this experimental study, sixty-eight primary lateral incisors were randomly allocated in four groups (*n* = 17): Adper Single Bond 2 (ASB), Clearfil SE Bond 2 (CSE), G-Premio Bond in etch-and-rinse mode (GP-ER), and G-Premio Bond in the self-etch mode (GP-SE). The coronal one third of root canals was filled with resin composite. The push-out test was performed using a universal testing machine. ANOVA and LSD tests were used to analyze the data (*P* < 0.05).

**Results:**

One-way ANOVA showed significant differences between the four groups in push-out bond strength (*P* = 0.002). The ASB and GP-SE groups showed lower and higher bond strengths, respectively. The failure mode distribution did not differ between the bonding agents used (*P* = 0.763). Adhesive and mixed failures were more frequent.

**Conclusion:**

The GP-SE, GP-ER, and CSE exhibited significantly higher push-out bond strength than ASB. A universal adhesive system and 6th generation self-etch adhesives are recommended for use with resin composite posts in primary anterior teeth. Regarding the advantages of these bonding agents, such as fewer clinical steps, lower technical sensitivity, and easy application, they can be a good option for restoring primary teeth with short resin composite posts.

## 1. Introduction

Early childhood caries is among the main causes of early anterior tooth loss [1, 2] because it can destroy the coronal structure of the teeth [3]. Prior to the introduction of bonding agents, extraction was the only solution for severely decayed primary anterior teeth [4]. Today, most such teeth can be saved thanks to the advances in restorative materials.

Different techniques are employed to restore primary anterior teeth [5], like resin-modified glass ionomer, resin composites, zirconia, and celluloid crowns. The dentist should choose the best option for individual teeth based on the strengths and weaknesses of each material [6]

Resin composites are among the most commonly used materials for restoring primary teeth due to their high durability, optimal esthetics, favorable adhesion to the tooth structure, and easy application [5]. Extensive carious lesions in primary teeth often lead to pulpal involvement due to the short and small size of the primary tooth crowns. The small remaining tooth structure in such teeth makes it difficult to restore them with optimal retention and esthetics [2, 7].

The currently available bonding agents include the etch-and-rinse and self-etch systems. The number of application steps has decreased from three steps in the 4th generation bonding agents to one step in the 7th and 8th generations [3, 5].

Considering the simple clinical application steps, faster application, and lower technical sensitivity of the one-step self-etch adhesives, they can be beneficial for children, especially young children with poor cooperation. However, newer generations of these adhesive systems, known as universal adhesives, can be applied in total-etch, selective-etch, and self-etch modes [5, 8].

The density and diameter of dentinal tubules are greater in primary teeth. Also, the primary teeth are less mineralized and have limited available bonding surface compared with permanent teeth. All these factors have raised doubts about the bond strength and durability of restorations in primary teeth compared with permanent teeth [9, 10].

Considering the limitations of bond strength in primary teeth, in severely decayed incisors where pulpectomy is carried out, intracanal retention is necessary for the durability of resin composite restorations [11]. Several types of posts are available in pediatric dentistry, such as prefabricated posts, orthodontic wire cast posts with macro-retentive elements, reverse metallic posts, resin composite posts, fiber posts, and biologic posts. Resin composite posts are most commonly used for this purpose [3, 11].

Few studies [1, 3] have assessed the bond strength of adhesive systems in primary teeth using the push-out test. In the present study, the push-out test was performed to evaluate the bond strength of various adhesive agents.

Considering the small number of studies on the effectiveness of novel one-step adhesive systems for bonding intracanal resin composite posts in primary teeth, this study aimed to assess the push-out bond strength of resin composite posts to the intracanal dentin in primary teeth using different adhesive systems.

## 2. Materials and Methods

### 2.1. Ethical Aspects

This in vitro study was approved by the Research Ethics Committee of Yazd Shahid Sadoughi University of Medical Sciences (IR.SSU.REC.1398.043).

### 2.2. Study Design

Sixty-eight maxillary primary lateral incisors with approximately similar root canal diameters were included in this study. The teeth had been extracted because the parents did not want to have these teeth restored. The physiological resorption of the roots did not exceed one-fourth of the root length. Also, the root surface had no carious lesions, fractures, or cracks.

### 2.3. Specimen Preparations

The extracted teeth were stored in 0.5% chloramine T solution for one week after removing soft tissue residues. The teeth were stored in distilled water during the study period to prevent dehydration. The tooth crowns were sectioned 1 mm above the cementoenamel junction with a diamond disc (MTI, Germany) under water coolant.

### 2.4. Root Canal Preparation

The root canals were instrumented with #15 to #45 K-files (Diadent, Korea) using the step-back technique. Root canal irrigation was performed with saline solution after using each file. After drying the root canals with #45 paper points (Diadent, Korea), calcium hydroxide paste with iodoform (Metapex, Meta Biomed Co., Ltd., Chungbuk, South Korea) was injected into the canals with gentle pressure.

### 2.5. Post Space Preparation

To prepare the post space, the calcium hydroxide-iodoform paste was removed from the orifice till the 4 mm depth of root canal using a small spoon excavator (Dental Device, Tehran, Iran), and self-cured glass-ionomer cement (GC FUJI 2; GC Corporation, Tokyo, Japan) was applied over the calcium hydroxide paste with iodoform in a 1 mm thickness. Excess cement was removed from the walls of the post space using a small spoon excavator (Dental Device, Tehran, Iran).

### 2.6. Grouping

After preparing the teeth and the intracanal space, based on table of random numbers, the teeth were randomly assigned to four groups (*n* = 17) based on the bonding agent type:

1. Adper Single Bond 2 (3M ESPE, St. Paul, MN, USA) (ASB)
2. Clearfil SE Bond 2 (Kuraray Co., Osaka, Japan) (CSE)
3. G-Premio Bond (GC Corporation, Tokyo, Japan) in etch-and-rinse mode (GP-ER)
4. G-Premio Bond (GC Corporation, Tokyo, Japan) in self-etch mode (GP-SE)

### 2.7. Bonding Procedure

1. After conditioning the dentin surface with 37% phosphoric acid (Scotchbond Acid, 3M ESPE, St. Paul, MN, USA) for 15 s, the root canal was irrigated and dried for 10 s. The ASB adhesive (3M ESPE, St. Paul, MN, USA) was applied to the dentin using a micro-brush. It was then subjected to a gentle air stream for 5 s for solvent evaporation, and then the adhesive was light-cured for 20 s (according to the manufacturer's instructions).
2. After applying the primer on the root canal surface and waiting for 20 s, it was dispersed by a gentle air stream for 5 s. The bonding agent was then applied to the surface using a micro-brush. The primer and bonding agent were light-cured for 20 s.
3. Prior to the application of GP bonding (GC Corporation, Tokyo, Japan), the dentin surface was conditioned for 10 s; then, GP one-step universal adhesive (GC Corporation, Tokyo, Japan) was applied, thinned by gentle air flow for 5 s, and light-cured for 20 s.
4. GP bonding agent was applied to the dentin surface, subjected to gentle air flow for 5 s, and then light-cured for 20 s.

After preparing the root canals and applying the bonding agents in each group, resin composite (Z250; 3M ESPE, St. Paul, MN, USA) was incrementally applied in a maximum of 2 mm thick increments to create resin composite posts. Furthermore, each layer was separately cured for 40 s by an LED light-curing unit (Woodpecker, China) at a light intensity of 800 mW/cm^2^.

### 2.8. Sectioning

The root canal orifice was sealed with 2 mm of Z250 resin composite. The specimens were mounted in transparent acrylic blocks perpendicular to the longitudinal axis of the tooth (in the apicocervical direction). Next, 1 ± 0.05 mm thick slices were sectioned at the mid-coronal part of the root by a CNC cutting machine.

### 2.9. Push-Out Test Procedure

The push-out test was performed using a universal testing machine (K-21046, Water + Bai, Switzerland). The specimens were subjected to a force at a crosshead speed of 0.5 mm/min in an apicocoronal direction through a column-shaped tip using a 5 KN load cell applied to the center of the post until failure occurred. The diameter of the metal tip of the machine was 1 mm. The maximum force causing debonding in each specimen was recorded in Newtons (N). To report the bond strength in megapascals (MPa), the amount of force recorded in Newtons was divided by the cross-sectional area of the specimens (mm^2^).

Bond strength (MPa) = force (N)/cross-sectional area (mm^2^).

To calculate the cross-sectional area, all the specimens were photographed under a stereomicroscope (ZTX 3E, China) before performing the bond strength test. The apical and coronal surface areas of the root canal were calculated on the photographs of each specimen using the Motic Image Plus 3.0 software, and the cross-sectional area of the bonding surface was calculated using the following formula.:

Cross-sectional area (mm^2^) = 0.5 [coronal surface area (mm) + apical surface area (mm)] height.

### 2.10. Failure Mode Determination

After the bond strength test, the specimens' failure modes were determined under a stereomicroscope (ZTX 3E, China) at ×32 magnification.

The failure modes were categorized as adhesive, cohesive, and mixed.

### 2.11. Statistical Analyses

Statistical analyses were carried out using SPSS 17 (SPSS Inc Chicago, USA). The normality of data distribution was assessed by the Kolmogorov–Smirnov and Shapiro–Wilk tests. Since the data were distributed normally, they were analyzed with one-way ANOVA, followed by the LSD test for pairwise comparisons. In addition, Fisher's exact test was conducted to determine whether the fracture types changed according to the bonding agents (Table 1).

## 3. Results

One specimen from each CSE, GP-ER, and GP-SE group was excluded from the study due to a fracture during the preparation and testing process. Consequently, sixty-five specimens were examined.  Table 2 presents the push-out bond strength of specimens (MPa) in the four groups.

ANOVA showed significant differences in push-out bond strengths between the four groups (*P*=0.002). Therefore, the LSD test was applied for pairwise comparisons. Based on the findings, the ASB group had a significantly lower bond strength than the other groups (*P* < 0.05). ASB group showed significant differences from the other groups. The results of pairwise comparisons showed that ASB group has significant difference with CSE (*P*=0.019), GP-ER (*P*=0.001), and GP-SE (*P*=0.001) groups.

Table 3 presents the failure mode percentages. The fracture mode distribution did not differ in terms of the bonding agents used (*P*=0.763), and most failures were adhesive.

## 4. Discussion

The clinical durability of resin composite restorations depends on the adhesive system and its ability to achieve an efficient resin composite-dentin bond [3].

Various tests can be employed to evaluate the bond strength of intracanal resin composite posts to intracanal dentin, such as the shear, micro-tensile, pull-out, and push-out tests. The push-out test applies a shear force to the resin composite-adhesive and adhesive-dentin interface and provides results closer to the clinical setting [1].

Despite the widespread use of adhesive agents in pediatric dentistry, adequate information regarding the function of adhesive systems, especially the novel systems in primary teeth, is not available [8]. Also, the available studies on the adhesive systems have been mainly conducted on permanent teeth, with contradictory results [12]. Such variations in findings are attributed to morphological and structural differences between the primary and permanent teeth [13]. The lower concentration of calcium and phosphorus in peritubular and intertubular dentin [13] and the higher density and diameter of dentinal tubules in primary teeth than in permanent teeth facilitate acid penetration into the tubules, leading to the deeper penetration of acid and subsequent dentin demineralization [14]. Consequently, it is recommended to use milder acids or decrease the etching time to 15 s in primary teeth [1].

A comparison of the three types of adhesive systems in the present study showed significantly lower bond strength in the ASB group than in the other three groups. Moreover, CSE and GP used in both self-etch and etch-and-rinse modes showed acceptable bond strength values. Although the difference was not significant, GP-SE showed the highest bond strength, followed by GP-ER and CSE.

Memarpour et al. [15] reported that ASB had a lower bond strength to the coronal structure of primary teeth compared with Scotchbond Universal (in both self-etch and etch-and-rinse modes), which was consistent with the present study. In a study by Afshar et al. [3], ASB showed a bond strength comparable to CSE and Single Bond Universal. Kara et al. [1] indicated that the bond strength of ASB was similar to that of Futurabond M, which is a one-step self-etch system. However, Lenzi et al. [16] reported similar bond strength values of CSE and ASB, in contrast to the present study. This discrepancy in the results can be attributed to the differences in the applied adhesives [1, 3], the tests used, and dentin substrate (coronal dentin) [16].

The lower bond strength of ASB adhesive compared with self-etch adhesives can be attributed to a common mistake during the application of the 5th generation of adhesives, leading to the collapse of collagen fibrils during dentin drying in the etch-and-rinse protocol, which can prevent adequate penetration of resin monomers and subsequently decrease the bond strength. However, self-etch systems have lower technical sensitivity in this respect [17].

According to the literature, many factors affect the bond strength, including pH, solvent type, and adhesive filler content [3]. However, Kramer et al. [10] reported that pH was not an effective factor in the performance of adhesive systems. Self-etch adhesive systems applied in the present study have intermediate acidity, meaning they have a higher pH than the etch-and-rinse systems.

The present findings showed that GP had a similar performance in both self-etch and etch-and-rinse modes. Similar studies on coronal dentin by Memarpoor et al. [15] and Thanaratikul et al. [5] indicated that Scotchbond Universal and Single Bond Universal had similar bond strength in both application modes, confirming our results.

The etching effect of the self-etch systems is related to the acidic functional monomers, including 10-methacryloyloxydecyl dihydrogen phosphate (10-MDP) that reacts with the hydroxyapatite remnants along the collagen fibrils after etching, resulting in the interlocking of the adhesive in the tooth structure by simultaneous demineralization and monomer penetration [13, 18, 19]. Therefore, both micromechanical bonding (resin tag formation) and chemical bonding (MDP with calcium hydroxyapatite) are involved in the formation of the hybrid layer at the dentin-resin interface with MDP-containing adhesive systems [20]. The produced chemical salt has hydrophilic stability and can remain stable for a long time in aqueous environments [1]. As a result, the higher bond strength of GP and CSE can be attributed to the presence of MDP monomer in their composition.

Although no significant difference was observed in the bond strength of GP and CSE, GP had slightly higher bond strength, consistent with the finding of Kamble et al. [21], who showed that Futurabond DC, an 8th generation adhesive, had higher bond strength than Adper SE Plus, a 6th generation adhesive. In addition, GP showed a higher performance than the other two adhesives due to the presence of acetone in its composition; however, CSE and ASB are water/ethanol-based. Contrary to the results of the present study, Thanaratikul et al. [5] noted that Single Bond Universal had a lower performance than CSE due to the lower amount of MDP monomer in Single Bond Universal and its subsequently lower chemical bonding potential. In a study by Tsujimoto et al. [18], GP resulted in lower bond strength than CSE in permanent teeth; such discrepancy can result from morphological and structural differences between the primary and permanent teeth. Moreover, GP contains a 4-META monomer that forms covalent bonds with calcium and can effectively improve the bond strength [10]. However, the results of in vitro studies cannot be generalized to clinical conditions and should be confirmed by clinical trials. Another limitation of this in vitro study was that aging of specimens was not performed, and the performance of adhesives was not evaluated for a long time. Thus, future studies are required to address these shortcomings.

## 5. Conclusion

This study showed that universal bonding systems (self-etch and etch-and-rinse modes) can be used to bond resin composite posts to intracanal dentin of primary anterior teeth. Regarding the advantages of these bonding agents, such as fewer clinical steps, less technical sensitivity, and easy application, they can be a good option to restore primary teeth.

## Figures and Tables

**Table 1:** The characteristics and compositions of adhesive systems used in each experimental group (*n* = 17).

Groups	Adhesive	Adhesive type	Composition	Manufacturer
Group 1	Adper Single Bond 2	Total-etch self-priming	Etchant: 35% phosphoric acid Adhesive: Bis-GMA, dimethacrylate resins, HEMA, Vitrebond™ copolymer, filler, ethanol water, photo-initiators	3M ESPE, St. Paul, MN, USA

Group 2	Clearfil SE Bond	Self-etch 2-step	Primer: MDP, HEMA, hydrophilic dimethacrylate, N,N-diethanol p-toluidine, water Adhesive: MDP, Bis-GMA, HEMA, hydrophobic dimethacrylate, d1-comphorquinone, N,N-diethanol p-toluidine, silanized silicate	Kuraray Co., Osaka, Japan

Group 3	G-Premio Bond	Universal self-etch in etch-and-rinse mode (GP-ER)	Etchant: 35% phosphoric acid 10-MDP, phosphoric acid ester monomer, dimethacrylate, 4-MET, MEPS, acetone, silica, initiators	GC Corporation, Tokyo, Japan

Group 4	G-Premio Bond	Universal self-etch in self-etch mode (GP-SE)	10-MDP, phosphoric acid ester monomer, dimethacrylate, 4-MET, MEPS, acetone, silica, initiators	GC Corporation, Tokyo, Japan

**Table 2:** Mean push-out bond strengths of different adhesive systems to intracanal dentin of primary lateral incisors in the four groups.

Group	Number of specimens	Mean (MPa)	Standard deviation	95% CI		Lower	Higher
				Upper bound	Lower bound		
(1) ASB	17	6.596	4.772	4.143	9.051	0.95	16.13
(2) CS	16	10.172	3.951	8.066	12.277	2.73	17.11
(3) GP-ER	16	11.547	4.052	9.387	13.707	5.11	17.59
(4) GP-SE	16	11.942	4.178	9.715	14.168	4.09	18.32
Total	65	10.014	4.681	8.851	11.171	0.95	18.32

*P* value = 0.002 (ANOVA).

**Table 3:** The frequency percentage of bond failure modes of different adhesive systems to intracanal dentin of primary lateral incisors in the four groups.

Type of bonding	Type of fracture		
	Adhesive	Mix	Cohesive
ASB	13 (76%)	4 (24%)	0
CSE	9 (56%)	6 (37%)	1 (7%)
GP-SE	9 (56%)	6 (37%)	1 (7%)
GP-ER	9 (56%)	5 (31%)	2 (13%)

## Data Availability

The data are available upon request.
